# Predictors of moderate to severe obstructive sleep apnea in patients with lung cancer

**DOI:** 10.1186/s12931-024-02789-z

**Published:** 2024-05-07

**Authors:** Mohamed H. Zidan, Hany M. Shaarawy, Heba S. Gharraf, Suzan F. Helal, Maged Hassan, Rana Rizk

**Affiliations:** 1 Chest Diseases Department, Alexandria Faculty of Medicine, Khartoum Square, Alexandria, Egypt; 2 Pathology Department, Alexandria Faculty of Medicine, Alexandria, Egypt

**Keywords:** Lung cancer, OSA, Performance status

## Abstract

**Background and objectives:**

OSA is a known medical condition that is associated with several comorbidities and affect patients’ quality of life. The association between OSA and lung cancer remains debated. Some studies reported increased prevalence of OSA in patients with lung cancer. We aimed to assess predictors of moderate-to-severe OSA in patients with lung cancer.

**Methods:**

We enrolled 153 adult patients who were newly diagnosed with lung cancer. Cardiorespiratory monitoring was performed using home sleep apnea device. We carried out Univariate and multivariate logistic regression analysis on multiple parameters including age, gender, smoking status, neck circumference, waist circumference, BMI, stage and histopathology of lung cancer, presence of superior vena cava obstruction, and performance status to find out the factors that are independently associated with a diagnosis of moderate-to-severe OSA.

**Results:**

Our results suggest that poor performance status is the most significant predictor of moderate to severe OSA in patients with lung cancer after controlling for important confounders.

**Conclusion:**

Performance status is a predictor of moderate to severe OSA in patients with lung cancer in our population of middle eastern ethnicity.

## To the editor

Lung cancer is considered the most common cause of cancer-related deaths in males and females worldwide. The incidence and mortality of lung cancer may vary according to smoking status, gender, ethnicity, environmental exposures, and economic state. 11.4% of new cancer cases diagnosed in 2020 were lung cancers, making it the most common cancer in males [1].

Obstructive sleep apnea (OSA) is characterized by recurrent episodes of upper airway partial or total collapse leading to hypopnea or apnea, respectively. The prevalence of OSA vary widely worldwide. In population-based studies, OSA prevalence was estimated to be around 14-50% in males and 5-23% in females [2]. Intermittent hypoxemia and sleep fragmentation play an important role in the inflammatory state in OSA and are responsible for many consequences as cardiovascular complications, diabetes mellitus and metabolic syndrome, psychological problems including depression, reduced quality of life and cognitive dysfunction [3].

The association between OSA and lung cancer has been explored and several studies suggested that lung cancer increases the risk of OSA and that OSA enhance the occurrence of lung cancer [4, 5]. It was also suggested that severe OSA could increase mortality of lung cancer [5]. Intermittent hypoxemia and fragmented sleep were reported to be the main factors for the association between both diseases [4]. Both factors cause change in sympathetic tone, enhance inflammatory process and recruitment of more inflammatory cells and cytokines, increase oxidative stress and angiogenesis. This occurs through different pathways. The most important is hypoxia inducible factor (HIF-1) transcription factor that activates several genes. These genes are involved in many cellular processes as glucose metabolism, angiogenesis, survival, and cellular death. This HIF-1 is recognized as a biomarker that is associated with aggressiveness of cancer in OSA [6]. Another consequence of intermittent hypoxemia is enhanced tumor associated macrophages into tumor site. Also, it causes increase in transformation of macrophages from anti-tumor phenotype (M1) into tumor promoting one (M2) [7].

This is a prospective observational study which recruited patients with new lung cancer diagnosis and examined sleep-disordered breathing in the study cohort to construct a predictive model to assess the most important variables that are potentially associated with the occurrence of sleep disordered breathing in such population.

Over a 21-month period the study prospectively recruited patients with newly diagnosed lung cancer confirmed by tissue biopsy. Patients were identified from the Chest Clinic of a tertiary hospital. The study protocol was approved by the Ethics Committee at Faculty of Medicine, Alexandria university, Egypt (ref 0201426).

The study included adult patients with newly diagnosed lung cancer. The study excluded patients who had history of any malignancy, previous diagnosis of OSA, previously treated with continuous positive airway pressure (CPAP) or had significant hypoxemia.

Cardiorespiratory monitoring was done using ResMed Apnea Link Air™ device (ResMed Inc, San Diego, CA). The device allowed assessment of oxygen saturation and heart rate, airflow, and thoracic movement. Scoring was performed according to the American Academy of Sleep Medicine guidelines version 2.4 [8]. OSA was defined as apnea hypopnea index (AHI) ≥ 5 events/hour, and moderate-to-severe disease was defined as AHI ≥ 15 events/hour. Performance status was assessed using Karnofsky performance status scale [9]. Staging of lung cancer was done according to the International Association for the Study of Lung Cancer eighth edition of the tumor, node, and metastasis classification of lung cancer [10]. Classification of lung cancer was performed using the 2021 WHO classification of lung tumors [11].

Data were analyzed using SPSS software package version 20.0. **(**Armonk, NY: IBM Corp**)**. We carried out Univariate logistic regression analysis on multiple parameters (age, gender, smoking status, body mass index (BMI), neck circumference and waist circumference, performance status, Epworth sleepiness scale, presence of diabetes mellitus, presence of superior vena cava obstruction, stage, and histological type of lung cancer) to investigate their relationship with the presence of moderate-to-severe OSA. We then created multivariate logistic regression models for parameters with P value < 0.25 to find out the factors that are independently associated with a diagnosis of moderate-to-severe OSA. We used P value < 0.25 as the most commonly used cutoff value in medical statistics (P < 0.05) is often seen as too strict and leads to dropping of clinically significant variables. To that effect, we aimed to include all clinically relevant variables that reached a cut-off of 0.20 or 0.25 in univariable analyses as suggested by some statisticians [12].

The study included 153 patients newly diagnosed with lung cancer recruited from March 2021 to December 2022. The baseline characteristics (age, gender, pack year index (PYI), comorbidities, BMI, neck circumference, and waist circumference), lung cancer-related parameters (performance status, presence of superior vena cava obstruction, stage, and histological type of lung cancer) and sleep parameters (Epworth sleepiness scale, oxygen desaturation index (ODI), and time of oxygen saturation spent below 90% (T90%) during total sleep time), are summarised in Table 1. 104 patients had no-to-mild OSA, and 49 patients had moderate-to-severe OSA. There was a significant difference between the group of patients with no or mild OSA and the group with moderate-to-severe OSA of patients regarding ODI, T90%, and performance status, (Table 1).

On univariate regression, performance status showed association with a diagnosis of moderate-to-severe OSA (P value < 0.001) with adjusted odds ratio 0.776 (95% CI 0.710–0.849). A multivariate logistic regression model including BMI, neck circumference, waist circumference, Karnofsky performance status scale, stage of lung cancer, and presence of diabetes mellitus was created, and the only parameter that showed significant relation to moderate and severe OSA was karnofsky performance status scale (P value < 0.001) with adjusted odds ratio 0.777 (95% CI 0.710–0.851), (Table 2). Another regression model was constructed to compare patients with no OSA (*n* = 19) versus patients with any degree of OSA (*n* = 134). Performance status remained a significant predictor of OSA diagnosis (P value < 0.001), with neck circumference (P value = 0.037) and waist circumference (P value = 0.019) also emerging as statistically significant predictors of OSA diagnosis.

The results of this study suggest that poor performance status is the most significant predictor of moderate to severe OSA in patients with lung cancer after controlling for important confounders such as waist circumference, neck circumference and BMI.

Performance status is expected to be poor in patients with symptomatic lung cancer. This can be caused by cancer itself or any associated disease. This poor performance status will affect the choice of management plan for these patients [13]. Therefore, early detection of comorbid conditions that can worsen performance status, and hence lead to poorer outcomes, is a clinical priority. As treatment of those patients with OSA is reported to improve their sleep quality, performance status, and quality of life, it is important to offer the best treatment options for OSA [14].

As our study suggested, a performance status that appears to be worse than what would be expected from the patient’s comorbidities should be a reason to consider screening for OSA. This is particularly relevant in patients with advanced malignancy who will have lost weight due to the catabolic state of advanced malignancy, and thus may not fulfil the usual BMI bracket of typical OSA patients. To the best of our knowledge, performance status in patients with comorbid OSA and lung cancer was not reported in previous studies.

The limitation of this study is the relatively small sample size with no healthy control subjects included. Also, we used level 3 sleep study for diagnosis of OSA which can’t differentiate between wake and sleep states and limits the use of arousal criteria for diagnosis of hypopnea. Finally, the majority of the included patients had advanced lung cancer and therefore the conclusions are less relevant to patients with early-stage cancer.

In conclusion, performance status is a predictor of moderate to severe OSA in patients with lung cancer in our population of our middle east ethnicity.

**Table 1:** Comparison between non-OSA/ Mild OSA and moderate/ severe OSA according to different parameters

	Normal/ Mild OSA (*n* = 104)	Moderate/ Severe OSA (*n* = 49)	Test of Sig.	*P*
**Age (/years); Mean ± SD**	59.7 ± 11.8	60.6 ± 9.5	t = 0.435	0.664
**Gender**				
Male	84 (80.8%)	36 (73.5%)	χ^2^ = 1.049	0.306
Female	20 (19.2%)	13 (26.5%)		
**PYI; Median (IQR)**	40 (20–60)	40 (0–60)	U = 2501.0	0.853
Non-smoker	22 (21.2%)	13 (26.5%)	χ^2^= 0.546	0.460
Smoker	82 (78.8%)	36 (73.5%)		
**Associated comorbidities**				
DM	26 (25%)	18 (36.7%)	χ^2^ = 2.239	0.135
HTN	29 (27.9%)	17 (34.7%)	χ^2^ = 0.734	0.391
Cardiac or renal problems	9 (8.7%)	3 (6.1%)	χ^2^ = 0.295	^FE^*p*=0.753
Morbid obesity	2 (1.9%)	0 (0%)	χ^2^ = 0.955	^FE^*p*=1.000
Others	8 (7.7%)	1 (2%)	χ^2^ = 1.922	^FE^*p*=0.273
**BMI (kg/m** ^**2**^ **); Mean ± SD**	25.1 ± 5.5	26.8 ± 4.7	t = 1.968	0.051
Underweight (< 18.5)	11 (10.6%)	2 (4.1%)	χ^2^= 3.223	0.359
Normal weight (18.5–24.9)	42 (40.4%)	19 (38.8%)		
Overweight (25–29.9)	40 (38.5%)	19 (38.8%)		
Obese (≥ 30)	11 (10.6%)	9 (18.4%)		
**Neck circumference; Mean ± SD**	37.9 ± 2.8	38.4 ± 2.4	t = 1.135	0.258
**Waist circumference; Mean ± SD**	94.2 ± 14.1	97.7 ± 12.6	t = 1.504	0.135
**Epworth sleepiness scale; Median (IQR)**	0 (0–0)	0 (0–3)	U = 2243.0	0.132
**ODI; Median (IQR)**	7.1 (4–9.4)	19.4 (14–24.1)	U = 247.0^*^	< 0.001^*^
**T90% ; Median (IQR)**	1.7 (0–10)	6 (2–22)	U = 1738.0^*^	0.001^*^
**Karnofsky performance status scale (%) ;Median (IQR)**	50 (40–60)	30 (30–40)	U = 449.0^*^	< 0.001^*^
**Stage**				
I	1 (1%)	0 (0%)	χ^2^= 2.855	^MC^p= 0.405
II	8 (7.7%)	2 (4.1%)		
III	26 (25%)	8 (16.3%)		
IV	69 (66.3%)	39 (79.6%)		
**Presence of SVCO**	6 (5.8%)	1 (2%)	χ^2^ = 1.061	^FE^*p*=0.431
**Histopathology**				
SCLC	17 (16.3%)	9 (18.4%)	χ^2^ = 0.096	0.756
Adenocarcinoma	43 (41.3%)	22 (44.9%)	χ^2^ = 0.172	0.678
SCC	42 (40.4%)	18 (36.7%)	χ^2^ = 0.186	0.666
Undifferentiated malignant tumor	2 (1.9%)	0 (0%)	χ^2^ = 0.955	^FE^*p*=1.000

*Abbreviations* PYI; pack year index, BMI; body mass index, ODI; oxygen desaturation index, SVCO; superior vena cava obstruction, T90%; time of oxygen saturation spent below 90% during total sleep time, SCLC; small cell lung cancer, SCC; squamous cell carcinoma

t: Student t-test, U: Mann Whitney test, χ^2^: Chi square test, MC: Monte Carlo, FE: Fisher Exact p: p value for comparing between Normal/ Mild OSA and Moderate/ Severe OSA

* Statistically significant at *p* ≤ 0.05

**Table 2:** Univariate and multivariate logistic regression analysis for the different parameters to discriminate between Moderate/ Severe OSA from Normal/ Mild OSA (*n* = 49 vs. 104)

	Univariate		^#^Multivariate	
	*p*	OR (LL – UL 95%C.I)	*p*	OR (LL – UL 95%C.I)
**Age (/years)**	0.662	1.007 (0.976–1.039)		
**Female**	0.308	1.517 (0.681–3.375)		
**PYI**	0.582	0.996 (0.984–1.009)		
**BMI**	0.054	1.067 (0.999–1.139)	0.740	1.030 (0.865–1.226)
**Neck circumference**	0.258	1.076 (0.948–1.220)	0.943	1.008 (0.815–1.247)
**Waist circumference**	0.137	1.019 (0.994–1.044)	0.965	1.001 (0.940–1.067)
**Karnofsky performance status scale (%)**	< 0.001^*^	0.776 (0.710–0.849)	< 0.001^*^	0.777 (0.710–0.851)
**Presence of SVCO**	0.325	0.340 (0.040–2.907)		
**Increase in Stage**	0.094	1.700 (0.913–3.165)	0.339	1.569 (0.623–3.954)
**Histopathology**				
SCLC	0.756	1.151 (0.473–2.805)		
Adenocarcinoma	0.678	1.156 (0.583–2.293)		
SCC	0.666	0.857 (0.425–1.727)		
Undifferentiated malignant tumor	0.999	–		
**ESS**	0.481	1.029 (0.951–1.112)		
**DM**	0.137	1.742 (0.839–3.618)	0.542	1.415 (0.463–4.327)

*Abbreviations* PYI; pack year index, BMI; body mass index, SVCO; superior vena cava obstruction, SCLC; small cell lung cancer, SCC; squamous cell carcinoma, ESS; Epworth sleepiness scale, DM; diabetes mellitus

OR: Odd`s ratio, C.I: Confidence interval, LL: Lower limit, UL: Upper Limit

^#^All variables with *p* < 0.25 was included in the multivariate * Statistically significant at *p* ≤ 0.05

## Data Availability

All data generated or analyzed during this study are included in this article. Further enquiries can be directed to the corresponding author.
